# Reviewing the Environmental and Human Health Knowledge Base of Carbon Nanotubes

**DOI:** 10.1289/ehp.9652

**Published:** 2007-05-10

**Authors:** Aasgeir Helland, Peter Wick, Andreas Koehler, Kaspar Schmid, Claudia Som

**Affiliations:** 1 Technology and Society Lab, EMPA (Swiss Federal Laboratories for Materials Testing and Research), St. Gallen, Switzerland; 2 Institute for Human-Environment Systems, ETH (Swiss Federal Institute of Technology) Zurich, Zurich, Switzerland; 3 Laboratory for Biocompatible Materials, EMPA, St. Gallen, Switzerland; 4 Institute for Occupational Health Sciences, Lausanne, Switzerland

**Keywords:** carbon nanotubes, cytotoxicology, ecotoxicology, environment, environmental fate, human health, *in vitro*, *in vivo*, nanomaterials, nanotechnology, nanotoxicology

## Abstract

Carbon nanotubes (CNTs) are considered one of the most promising materials in nanotechnology, with attractive properties for many technologic applications. The different synthesis, purification, and postprocessing methods produce CNTs with different physical characteristics, which can be applied in different fields ranging from composite materials, medical applications, and electronics to energy storage. The widespread projected use of CNTs makes it important to understand their potential harmful effects. In this environmental health review we observed a remarkable range of results of some of the toxicology studies. The comparability should be improved by further standardization and introduction of reference materials. However, at present the findings of this review suggest several key points: *a*) there are different types of CNTs, and therefore they cannot be considered a uniform group of substances; and *b*) in environmental compartments, CNTs can be bioavailable to organisms. The properties of CNTs suggest a possible accumulation along the food chain and high persistence. In organisms the absorption, distribution, metabolism, excretion, and toxicity of CNTs depend on the inherent physical and chemical characteristics such as CNT functionalization, coating, length, and agglomeration state that are influenced by the external environmental conditions during CNT production, use, and disposal stages. Characterized exposure scenarios could therefore be useful when conducting toxicologic studies. However, CNTs produce a toxic response upon reaching the lungs in sufficient quantity; this reaction is produced in a time-and dose-dependent manner. The identification of possible risks to human health and environment is a prerequisite for a successful introduction of CNTs in future applications.

The worldwide funding devoted to nanotechnology research and development by governments, industry, and venture capitalists was estimated to be around US$9.6 billion in 2005 (Lux Research Inc. 2006). A large portion of this spending is still being allocated to the development of nanoparticulate materials because of their many novel physical and chemical properties raising high expectations for a variety of applications. One of these new materials is carbon nanotubes (CNTs), which have commercial expectations in different manufacturing sectors.

However, epidemiologic studies of air pollution suggest that particulate matter has a strong association with cardiopulmonary diseases (Pope et al. 2004). Research has shown that nanoparticles may enter the human body more easily and be more biologically active because of their larger surface area per mass unit compared with that of larger particles (Oberdörster G et al. 2005). The prospective widespread use of engineered nanoparticles in consumer products may increase environmental, occupational, and public exposures dramatically. Consequently, different stakeholders have raised serious concerns regarding health effects of engineered nanoparticles (Helland et al. 2006). Recent review articles on the toxicity potential of nanoparticles (Nel et al. 2006; Oberdörster G et al. 2005) conclude that the toxicity of nanoparticles depends on specific physiochemical and environmental factors. Thus, the toxic potential of each type of nanoparticle has to be evaluated individually.

Here we review the currently available literature on the potential risks of CNTs to human health and the environment. We also investigated the life cycle of CNTs, as release into different environmental compartments may occur at the production stages as well as at the product’s use and disposal stages, which may directly or indirectly lead to human exposure. However, the published literature revealed many unanswered questions. Therefore, we also systematically interviewed seven leading world-class scientists and integrated their contemporary knowledge into this review (see Supplemental Material; http://www.ehponline.org/docs/2007/9652/suppl.pdf). This assisted us in identifying questions and developing recommendations. The scientists interviewed were key authors or project leaders who have investigated and reported the potential impacts of CNTs on human health or environment. Through this combined approach we are able to present an updated and contemporary knowledge base for scientific discussion.

In this review, we use the term “carbon nanotubes” when addressing the general aspects of the material, which includes single-walled carbon nanotubes (SWCNTs) and multiwalled carbon nanotubes (MWCNTs). Here “multi” is defined as two or more walls. The terms “SWCNTs” and “MWCNTs” are used in a specified manner.

## Exposure to Carbon Nanotube Material

### Exposure in occupational settings

Procedures for the handling of CNTs can result in aerosol release of these materials into the surroundings (Maynard et al. 2004). MWCNT aerosols generally have diameters between 20 nm to > 200 nm, lengths from 1,000 nm to > 10^6^ nm, and different shapes (straight, partly rigid, bent, curled, and partly flexible) that may appear single or in clumps or ropes (Donaldson et al. 2006).

Only one published study has investigated the potential for SWCNTs to become airborne. A laboratory study by Maynard et al. (2004) investigating the physical nature of the aerosol formed during mechanical agitation was complemented by a field study of SWCNT release during handling of unrefined SWCNTs. The authors found that sufficient agitation of unrefined SWCNT material can release fine particles into the air, but the concentrations generated while handling the material in the field were very low (< 53 μg/m^3^). The laboratory study also revealed that different SWCNT production methods produced different types of aerosols. The laser ablation process generated a more compact aerosol that was difficult to break down into smaller particles, whereas the HiPCO (high-pressure carbon monoxide) process generated a more extended material that was easier to break down into smaller particles and appeared to lead to higher airborne concentrations.

Maynard et al. (2004) also found glove deposits of SWCNTs during handling that were estimated at between 0.2 and 6 mg per hand and thus concluded that large SWCNT-containing clumps had the propensity to become airborne and could remain so for long periods. This may cause dermal exposure and health risks even in less well-protected areas. Maynard et al. (2004) noted that production volume was very small (research facility) and that workers took great care to reduce product loss during handling of the material. However, CNTs contain catalyst metals such as nickel, which is associated with increased risks of cancer in the nose region (Feron et al. 2002).

Cleaning operations can also lead to emissions. The cleaning of the production chambers is performed usually using solvents or water, tissues, brushes, and sponges that are discarded after cleaning (VDI 2004). This waste carries CNTs into the waste stream, thereby possibly becoming a source of release into the environment.

### Exposure through environmental media

Exposure through environmental media is highly relevant for several reasons: *a*) The widespread applications envisioned for CNTs may lead to substantial production volumes, and consequently to increased emissions into the environmental compartments air, groundwater, and soil. *b*) The physical and chemical processes in the environmental compartments may alter the properties of CNTs, for example, abiotic factors such as ultraviolet light may alter the coatings of CNTs as observed with fullerenes (Kamat et al. 1998) and quantum dots (Derfus et al. 2004). Consequently, this may also change the behavior of CNTs in the environment and thus influence their environmental fate and impact. *c*) CNTs are possibly one of the least biodegradable man-made materials ever devised (Lam et al. 2004). They are totally insoluble in water in pristine form (Lam et al. 2004) and are lipophilic by nature (Wu et al. 2006). It is generally known that biopersistent and lipophilic chemicals may accumulate along the food chain; therefore, such a scenario should also be evaluated with CNTs.

In aqueous environments, SWCNTs clump together to form aggregates in the micrometer range; these aggregates do not change in size distribution with increasing salinity or temperature (Cheng and Cheng 2005). However, the aggregation differs with pH changes in water (Cheng and Cheng 2005) and postsynthesis treatment of the SWCNTs with, for example, acid or surfactants (Chen et al. 2004). Both these studies found that pristine nanotubes formed stable aggregates, whereas acid-treated nanotube suspension showed greater dispersion variability over time, yielding looser structures at large-length scales and more compact structures at small-length scales. The addition of a surfactant to CNTs resulted in a hydrophilic interface at the tip of the nanotubes that significantly enhanced nanotube dispersion. In laboratory assessments designed to assess the potential migration in natural porous media, SWCNTs have been shown to have mobility and deposition behaviors different from those of other nanoparticles (Lecoanet and Wiesner 2004; Lecoanet et al. 2004). SWCNTs functionalized to facilitate dispersion in water displayed the highest mobility together with water-soluble fullerol, whereas colloidal C_60_, anatase titanium dioxide (TiO_2_), and ferroxane were among the least mobile of the nanomaterials evaluated.

The large surface area of CNTs may cause other molecules to adhere and potentially pick up pollutants and transport these throughout the environment (Kleiner and Hogan 2003). Several studies have investigated different carbon nanomaterials as superior sorbents of organic pollutants, metals, fluoride, and radionuclide 243-americium [^243^Am(III)] (Fiorito et al. 2006; Jia et al. 2005; Yang et al. 2006). Yang et al. (2006) found a high adsorption capacity of polycyclic aromatic hydrocarbons (PAHs) with different types of CNTs (MW and SW). This finding indicates a potential effect on the fate of PAHs upon their release into the environment. The adsorption capacity was found with the order SWCNTs > MWCNTs > C_60_ and seemed to be related to the surface area, micropore volume, and the volume ratios of mesopore to micropore.

Oberdörster et al. (2006), in a preliminary study, investigated the ingestion of SWCNTs by the suspension-feeding worm *Caenorhabditis elegans.* SWCNTs moved through the digestive tract and were not absorbed by the animal. However, even if SWCNTs did stay in the digestive tract, these materials could move up the food chain, as these worms and other organisms are consumed by benthivores. SWCNTs have also been shown to be bioavailable to aquatic organisms, as both water-solubilized (wrapped with a synthetic peptide) and unsolubilized SWCNTs were detected in the fecal material collected from the digestive tract in the exposed fish (Oberdörster E et al. 2005). For the water-solubilized SWCNT-exposed fish, clumps of SWCNTs were also found on the gill, but similar clumps were not visible in unsolubilized SWCNT-exposed fish. However, the fish mistook the unsolubilized SWCNTs (floating on top of the water) for food and ingested them (Oberdörster et al. 2006). Furthermore, because pristine CNTs are lipophilic, there is concern that they might be taken up by microbial communities and roots (Oberdörster et al. 2006) and, consequently, accumulate in plant tissues.

Carbonaceous nanoparticles, including MWCNTs, can also be formed by natural processes (Velasco-Santos et al. 2003) and anthropogenic combustion processes (Murr et al. 2004). Although these MWCNTs can be prime suspects in the pathogenesis of cardiopulmonary diseases induced by fine particulate matter, there are physical differences between combustion-generated and manufactured MWCNTs (Lam et al. 2006). These MWCNT structures may therefore be less important when the impacts of engineered CNTs are being assessed, as the studies to date suggest that when the properties of CNTs are altered by engineering, changes in the environmental fate of and human exposure to CNTs occur through the different environmental media.

## Human Health

### In vivo studies

#### Pulmonary toxicity

The first *in vivo* study of fullerene soot containing CNTs did not find any inflammation in the respiratory tract of guinea pigs or change of pulmonary function 4 weeks after exposure (Huczko et al. 2001), but it is possible that the method applied in this study (not examining lung pathology) did not reflect the actual toxicity of the material. The followup study with analysis 90 days postexposure of six different types of MWCNTs administered in individual doses (12.5 mg) to guinea pigs found small differences between the types of MWCNTs (Grubek-Jaworska et al. 2006; Huczko et al. 2005). However, for all types of MWCNTs there were multifocal granulomas observed around the materials, inflammatory reactions of terminal and respiratory bronchioles, and in some animals, mild fibrosis in alveolar septa.

SWCNT soot generated by the laser ablation method and intratracheally instilled in rats produced transient inflammation, cell injury effects, and a subsequent non-dose-dependent series of multifocal granulomas (Warheit et al. 2004). In comparison, equal doses of quartz produced sustained pulmonary inflammation, cytotoxicity, and fibrosis in a dose-dependent fashion. The authors questioned whether the failed observation of a dose-dependent relationship between SWCNT exposure and formation of the granulomas could be the result of the method of instillation and suggested that it should be clarified in an inhalation toxicity study.

In an intratracheal instillation study of mice, three types of SWCNTs were investigated (Lam et al. 2004)—two types made by the HiPCO method and one by the electric arc method. The results from all three types showed that regardless of the amount of metal impurities, dose-dependent lung lesions were characterized chiefly by interstitial granulomas. The study also showed that both SWCNTs and ultrafine carbon black were taken up by alveolar macrophages, but that the effects of these materials were different. Macrophages containing carbon black were homogeneously distributed over the alveolar space, but macrophages containing SWCNTs clustered to form granulomas in centrilobular locations. In comparison, quartz induced mild to moderate pulmonary inflammation, which was considered less severe than that induced with SWCNTs.

The consequent agglomeration of CNTs into nanoropes (Tagmatarchis and Prato 2004) makes it difficult to manipulate and administer these particles to experimental animals without forming nonrespirable particles that can lead to mechanical blockage of the airways, as observed in other studies (Grubek-Jaworska et al. 2006; Warheit et al. 2004). Therefore, Muller et al. (2005) compared the pulmonary toxicity of ground and unground MWCNTs in rats, using asbestos (Rhodesian chrysotile) and carbon black as references. The length of the individual MWCNT was reduced from 5.9 to 0.7 μm as a result of the grinding procedure. The distribution in the lungs after intratracheal instillation was different for the two types. Agglomerates of intact MWCNTs remained in the largest airways, whereas ground MWCNTs were much better dispersed on the lung tissue surface. Regarding toxicity, the study found that after 60 days there were indications of a higher degree of pulmonary inflammation with ground MWCNTs than that found with intact MWCNT-treated animals. The induced effects were dose dependent [bronchoalveolar lavage fluid (BALF) lactate dehydrogenase (LDH) activity and protein content]. Histologic and biochemical analyses demonstrated a fibrotic reaction (granulomas) in the bronchi with unground MWCNTs and in the alveolar space or interstitial tissue with ground MWCNTs. In summary, the findings of Muller et al. (2005) indicate that asbestos-ground MWCNTs and unground MWCNTs induce at least partially similar effects [inflammation, fibrotic reaction, and increased tumor necrosis factor (TNF)-α production], whereas carbon black showed a transient inflammation with elevated TNF-α. The authors therefore concluded that these data point to a specific toxicity related to the unique properties of CNTs.

In a pharyngeal aspiration study of mice by Shvedova et al. (2005), the effects of HiPCO-produced SWCNTs purified to a carbon content > 99% were investigated. The SWCNT aggregate depositions were correlated with granulomatous inflammation, whereas interstitial fibrosis with alveolar wall thickening was observed to be greater at 60 days than at 28 days postexposure in lung regions distant from the SWCNT aggregates. SWCNTs were compared with equal doses of nanoparticulated carbon black and fine crystalline silica, which did not induce granulomas and alveolar wall thickening and caused significantly less inflammation. Furthermore, the authors investigated whether exposure caused damage to pulmonary cells. This was confirmed by an increased number of alveolar type II cells (type II cells replicate after alveolar type I cell death). Exposure to SWCNTs also resulted in accumulation of an oxidative stress biomarker (4-hydroxynonenal) 1 day after exposure and also in a time- and dose-dependent depletion of a major antioxidant, glutathione, which was most severe 1 day postexposure. The rapid and dose-dependent fibrogenic response in regions of the lungs distant from SWCNT aggregates and in the absence of persistent inflammation was a unique finding in this study.

Initially, Shvedova et al. (2005) associated the interstitial fibrosis with deposition of dispersed SWCNT structures and pointed out that the mechanism for this response differs from the classic fibrogenic particle response in that it is not driven by chronic inflammation and chronic activation of alveolar macrophages. Additionally, the authors found that if pathogenic bacteria were inhaled by mice in combination with SWCNTs, bacterial clearance from the lungs was significantly slower. This indicates that SWCNTs, in addition to their primary effects, may also diminish general resistance to pathogenic attacks.

#### Dermal toxicity

There is only one published study *in vivo* on the dermal toxicity of fullerene soot containing CNTs (Huczko and Lange 2001). Forty volunteers were subjected to a patch test, and four albino rabbits were subjected to an eye test. The study found no evidence of the induction of any response; thus, the authors concluded that soot containing CNTs is not associated with any risks (Huczko and Lange 2001). However, information is insufficient on CNT material characterization and the study design for adequate validation of these results.

#### Translocation

There are some contradictory findings on the kinetics of water-soluble functionalized and radioactively labeled CNTs in the body. Wang et al. (2004) showed that when hydroxylated SWCNTs with radioactive iodine-125 atoms were injected into mice, the SWCNTs behaved like small molecules, passed easily through a number of compartments, accumulated especially in bone, and were distributed throughout the whole mouse body except the brain. Eighty percent of the total dosing of the SWCNTs was excreted by the feces and urine after 11 days. In contrast, Singh et al. (2006) found no accumulation of chelated diethylentriaminepentaacetic and indium-111-labeled SWCNTs or MWCNTs in mice after 24 hr. The biological behavior of functionalized (f)-CNTs is therefore comparable to small molecules. They are removed from the blood through the renal excretion route. No toxic side effects or mortalities were observed, and the excreted f-SWCNTs and f-MWCNTs were intact (Singh et al. 2006). Both studies are valid only for functionalized tubes and cannot be interpolated for nonfunctionalized tubes because of their hydrophobic nature. The difference in the distribution in bone is probably because the functionalization of the tubes was not the same, which influenced behavior in the organism.

#### Histopathologic analysis *ex vivo.*

Sato et al. (2005) investigated the influence of different MWCNT lengths on the cytotoxicity of MWCNTs in the human THP-1 leukemia cell line *in vitro* and in subcutaneous tissue of rat *in vivo*. In a long-term assay *in vivo*, the authors observed 4 weeks after surgery that an increased inflammatory response was established only by the 825-nm MWCNTs, as indicated by the formation of granulation tissue. Most of the 220-nm MWCNTs were observed in phagocytes, with many of these recognizable in lysosomes. Conversely, the 825-nm MWCNTs were also observed in the intercellular space.

#### In vitro *studies*

The methodology of how the toxicity of CNTs is evaluated depends strongly on how CNTs are administered to the cells (i.e., homogenously dispersed or not, with or without surfactant, type and concentration of the surfactant). Furthermore, CNTs contain contaminants that may be bioactive per se. Often the exact methodology and CNT characteristics are not described, which makes it difficult to compare available data.

#### Pulmonary cytotoxicity

In an *in vivo* study performed with nanoparticles such as TiO_2_, Geiser et al. (2005) showed that these particles could be taken up by the lung, passed through the air-blood barrier, and translocated into the bloodstream. This pathway cannot be excluded for CNTs and may be supported at the single-cell level. SWCNTs suspended in Pluronic F108 (a nonionic surfactant) were incorporated by peritoneal macrophage-like cells using near-infrared fluorescence microscopy. Cherukuri et al. (2004) found that 1.5 μg of 7.3 μg/mL SWCNTs were taken up within 24 hr by macrophage cell cultures grown in 24-well culture dishes. The fastest reaction evoked by SWCNTs in macrophages was an oxidative stress that occurred within hours (Kagan et al. 2006). The improved properties of nanomaterials also resulted in a catalytic activity. This catalytic activity may contribute to a new aggressive form of long-term toxicity. Oxidative stress is an imbalance between the production of reactive oxidizing species (ROS) and their degradation by antioxidants. The intracellular equilibrium may be disturbed by the presence and/or uptake of nanomaterials. The concentration of ROS may be increased by the particle itself or by the disturbance of the ROS degradation pathway. Both cause an additional production of ROS, which interacts uncontrollably with the cell membrane, DNA, and/or other cell compounds, severely damaging these cell compounds.

According to Muller et al. (2005) and as described in previous sections, the adverse effects of MWCNTs depend on the length of the material used *in vivo*. Similar to their *in vivo* study, the authors found that long untreated tubes using Triton X-100 as the vehicle evoked an LDH release of nearly 20% at a concentration of 100 μg MWCNTs/one 24-well of a 24-well plate, whereas the short ground MWCNTs induced a dose-dependent LDH release up to 35% greater than the corresponding vehicle (Triton X-100) treatment. This suggests that also *in vitro* the short MWCNTs are more toxic than the long ones. Interestingly, TNF-α was induced *in vivo* (measured in BALF) as well as *in vitro* (measured in preactivated peritoneal macrophages), indicating that TNF-α could be a reliable marker for future *in vitro* studies.

A comparative *in vitro* cytotoxicity study of several manufactured nanoparticles and nanotubes revealed that aggregated SWCNTs/ropes, MWCNT raw material, and MWCNT-aggregated tubes suspended in 5 μg/mL dimethylsulfoxide (DMSO) affected cell viability in murine lung alveolar macrophages at nearly similar concentrations (Murr et al. 2005). Compared with different oxide particles of differing quality such as aluminum oxide, zirconium oxide, or ferric oxide, the SWCNTs and MWCNTs expressed comparable median effective concentration (EC_50_) values (micrograms per milligrams), which is analogous to the same values obtained with asbestos used in the same studies as reference material (Murr et al. 2005; Soto et al. 2005). However, it is speculative to generalize these results in terms of health risks to humans, as the presented comparison study is based only on short-term effects during a 48-hr period. The physiologic relevance of these results remains to be determined.

In addition to the size, shape, and novel physical and chemical properties, the degree and type of agglomeration are important factors in the cytotoxicologic assessment of CNTs. Well-dispersed SWCNTs were less cytotoxic than micrometer-sized agglomerates of SWCNTs (Wick et al. 2007). To determine which carbonaceous nanomaterials have the most severe effect, comparison studies were conducted with the same biological model system. Jia et al. (2005) compared SWCNTs, MWCNTs, and C_60_ fullerenes suspended in cell culture medium and found that the cytotoxicity is apparently based on SWCNTs > MWCNT > quartz > C_60_. Fiorito et al. (2006) in a second study on murine and human macrophages claimed that graphite had the most severe effect, followed by SWCNTs and C_60_ fullerenes.

#### Dermal cytotoxicity

To emphasize the dermal toxicity of CNTs, Monteiro-Riviere et al. (2005) exposed human epidermal keratinocytes to MWCNTs that were produced without further purification processes. These particles were taken up by the keratinocytes in vacuoles where MWCNTs retained their structure, as demonstrated by transmission electron microscopy analysis. The cell viability parameters were reduced by 400 μg/mL MWCNTs to as much as 20% after a 24-hr exposure. The expression of interleukin (IL)-8 was increased up to 6 times (400 μg MWCNTs/mL) in a dose-dependent manner after that period compared with the corresponding control cell cultures (Monteiro-Riviere et al. 2005).

A detailed characterization of the molecular mechanism evoked by exposure of human skin fibroblast cells to 0.6 and 0.06 μg MWCNTs/mL was evaluated by gene microarray analysis. This analysis showed that MWCNTs induced cell cycle arrest and increased apoptosis and necrosis. Of the genes evaluated, 216 genes changed their expression levels after treatment with MWCNTs at 0.6 μg/mL. The most significant gene categories were Golgi vesicle transport, protein metabolism, secretory pathway, fatty acid biosynthesis G_1_/S transition of mitotic cell cycle, and cell homeostasis. The fact that these cellular processes were affected by the presence of MWCNTs indicates that this stress has a remarkable influence on cell performance (Ding et al. 2005).

Shvedova et al. (2003) exposed human keratinocytes to HiPCO-produced SWCNT material containing 30% by weight of iron. One of the first reactions on SWCNT treatment, which occurred 18 hr after incubation, was an increased oxidative stress, as indicated by the presence of free radicals. The decrease of the total antioxidant reserve and the reduction of vitamin E as well as the increase of the lipid peroxidation products compared with the control cell culture support strongly the presence and the damage of the oxidative stress within the cell cultures. Shvedova et al. (2003) claimed that this oxidative stress was the reason for the reduced cell viability.

In contrast to the work presented previously in which CNTs were suspended by sonification in the presence of a solvent or cell culture medium, Sayes et al. (2006) functionalized purified SWCNTs before dispersing them in the cell culture medium. They produced SWCNT–phenyl–sulfate, SWCNT–phenyl–sodium sulfate (SO_3_H), and six mixed SWCNT–carbon–sulfate and SWCNT–phenyl–SO_3_H samples with ratios of 18, 41, and 80, respectively. According to the authors only water-dispersible SWCNTs were of considerable interest for biological applications. The suspensions obtained were used to treat human dermal fibroblast cultures. The pristine unmodified SWCNTs evoked a rate of 80% cell death at a concentration of 20 μg/mL, whereas functionalized SWCNT–phenyl–SO_3_H induced < 5% cell death at a concentration of 2,000 μg/mL. Cytotoxicity was also strongly dependent on the type of sidewall groups: SWCNT–phenyl–SO_3_H was less toxic than the SWCNT with –phenyl-(COOH)_2_ group. The general conclusion reached by Sayes et al. (2006) was that an inverse relationship exists between the toxic potential and the degree of side wall functionalization. This is an important issue for further applications of CNTs in nanomedicine. The intensive skin cell–CNT interaction studies emphasize that penetration of CNTs is still not understood.

#### *In vitro* studies with neurons

Poly*m*-aminobenzene sulfonic acid and ethylenediamine functionalized MWCNTs (MWCNT–PABS; MWCNT–EN) coatings were used to coat glass cover slips as substrates for neuronal growth. To achieve this coating, the tubes were suspended in water and glass cover slips were coated with the suspension. After the water was evaporated, hippocampal neurons were seeded on the cover slips. After 3 days of incubation the number of branches per neuron, total neurite length, and the number of neurites per neuron of the cultivated neurons were increased on functionalized MWCNTs but not on pristine MWCNTs. However, it must be noted that the performance of the neurons grown on polyethyleneimine (positive control) was better than that of neurons grown on MWCNT-coated surfaces. Additionally, Hu et al. (2004) claimed that the surface charge of the modified MWCNT can be used to control neurite outgrowth. An increase in neuronal outgrowth and branching was achieved by the functionalization of SWCNTs with polyethyleneimine (SWCNT–PEI) compared with the modified MWCNTs (Hu et al. 2005). The authors described the morphology of these nerve cells in detail, but the functional proof, namely, in how far these cells exhibited spontaneous action potential and/or were able to be stimulated, is still lacking. All these studies reflected a positive effect of SWCNTs on neurons, but compared with other cytotoxicologic assessments, these tubes were immobilized on glass. A similar study was conducted by Liopo et al. (2006), who assessed growth and attachment of the neuroblastoma glioma NG108, a model neuronal cell, on unmodified SWCNT substrates and/or substrates from SWCNTs modified with 4-benzoic acid or 4-*tert*-butylphenyl functional groups using a simple functionalization method. Liopo et al. (2006) found that SWCNT films support cell growth but at a reduced level compared with that of tissue culture–treated polystyrene.

Therefore, all studies mentioned here investigated in principle the degree to which nanostructured surfaces with chemical characteristics of CNTs affect the outgrowth of nerve cells and not the toxicity of suspended CNTs.

#### *In vitro* cytotoxicity studies in different cell types

For human embryo kidney (HEK293) cells it was reported that in 0.5% DMSO, suspended SWCNTs are able to inhibit cell proliferation and to decrease cell adhesive ability in a dose- and time-dependent manner (Cui et al. 2005). Additionally, these HEK293 cells exhibit an active response to SWCNTs such as the secretion of a 20- to 30-kDa protein with unknown function. After SWCNT treatment, further observations were made that were analogous to those in the reports in the previous sections. These included observations on cell cycle arrest in the G_1_ phase, up- or down-regulation of cell cycle–associated genes, and formation of apoptosis/necrosis. The degree to which the presence of the DMSO affects the cell surface and cell performance is unclear in the present study.

As described previously, the surface chemistry strongly affects the toxic potential of CNTs. For example, the cell viability of human T lymphocytes was decreased to 80% by exposure to 400 μg/mL of oxidized MWCNTs (oxidized by refluxing the MWCNTs in concentrated nitric acid). The reduction in cell viability was explained by the increase of apoptotic cells after the treatment (Bottini et al. 2006). In comparison pristine MWCNTs reduced viability by up to 40% and carbon black by only 15% at the same concentration. Interestingly, the oxidized MWCNTs appeared to be shorter and straighter.

Several studies were conducted in which SWCNTs were modified by adding various biological molecules such as phospholipids conjugated with polyethylene glycol (PEG)–folic acid, PEG–NH_2_, PEG–S–S-DNA, etc. (Kam et al. 2004, 2005a, 2005b). In all the studies the water dispersibility increased and the tubes were incorporated in HL60 and HeLa cells. Depending on the modification of the SWCNTs, these tubes could act as multifunctional biological transporters, for example, DNA or siRNA (Bianco 2004; Bianco et al. 2005; Pantarotto et al. 2004). Unfortunately, no quantitative measurement was performed to allow a statement and comparison of these modified SWCNTs regarding the uptake rate and induced toxicity.

Carbon nanotubes have also been proposed by Price et al. (2004) as a possible new orthopedic/dental implant surface material because of their unique mechanical, electrical, and cytocompatibility properties. Cell viability and number of human osteoblast CRL-11372 were determined after 3, 6, 11, and 24 hr of incubation by ethidium homodimer and calcein AM staining. The authors used conventional fibers (diameter > 100 nm) and nanoscaled fibers (diameter < 100 nm). The fibers agglomerated within 1 week in cell culture media to ropes of about 340 nm in diameter in the case of conventional fibers and about 670 nm in the case of nanoscaled fibers. The nanoscaled carbon fibers appeared to influence osteoblast viability less than their conventional dimensioned counterparts. The suspended carbon fiber agglomerates were taken up by the osteoblasts and were incorporated in lysosomal vacuoles (Price et al. 2004). This future application of carbon nanotubes as implant material is in itself a promising issue, but one of the key questions is do carbon nanotubes influence the differentiation of osteoblast progenitor cells and to what degree is the formation and activation of osteoblasts affected?

In conclusion, CNTs were taken up by different cell types and evoked diverse effects in the cells. A first and fast reaction is the formation of free radicals (oxidative stress), which has been suggested as a key factor in further cell reactions [e.g., Nel et al. (2006)]. Several cell biological effects and changes in gene expression patterns were described, but because of the different CNT material and suspension procedures used, quantitative and comparison statements on the cytotoxicity of CNTs in different cell types and tissue are nearly impossible. Focused studies on inhalation exposures are needed to evaluate *in vitro* studies on their reliability and to build up the epidemiologic databases.

## Discussion

In the present review, we have investigated the state of knowledge regarding the impact of CNTs on human health and the environment. At the time of the review, there were approximately 50 studies focusing on the effects of CNTs on human health and environment, with the majority of them *in vitro* studies (Supplemental Material, Tables S4 and S5; http://www.ehponline.org/docs/2007/9652/suppl.pdf). Environmental impacts in particular have been investigated poorly. One of the findings of the expert interviews was that the current impact assessments have been conducted without any overall research strategy (Supplemental Material). Acknowledging this, the experts have called for a better coordination of research, which could provide interdisciplinary and complementary results.

**Figure 1:**
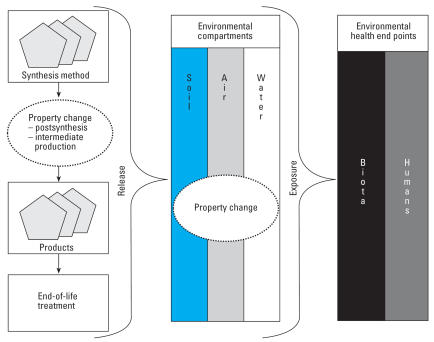
The life cycle and environmental fate of CNTs. CNTs may change properties during the life cycle of the product and in the environmental compartments. Humans and biota may therefore be exposed to different types of CNTs.

There are different types of CNTs produced in and applied to products with a variety of physical and chemical properties and potential exposure routes. The knowledge base suggests that altering these properties induces different effects on environmental health. Additionally, these properties may change during the life cycle of a product containing CNTs, as external physical and chemical influences differ at each stage. Therefore CNTs may cause different environmental health effects depending on the life cycle of the product and fate of the product in the environment. Tracing the fate of CNTs from each stage of the CNT product life cycle, as illustrated in Figure 1, may be an effective orientation for prioritizing research.

### CNT material

The particular properties of CNTs depend on the particular production process used (Supplemental Material; http://www.ehponline.org/docs/2007/9652/suppl.pdf). After synthesis the raw material contains nanoparticulate impurities that influence the toxicity of the species. Postsynthesis treatment alters various properties of CNTs such as length, purity, degree of aggregation, wall structure (doping), and surface functionalization. These properties are thought to determine toxicologic-relevant factors such as particle size, mobility in the environment, chemical reactivity, persistence, and bioavailability. Hence, the impacts of engineered CNTs are likely to differ from those of naturally occurring CNTs and may additionally depend on the technical application and circumstances of release.

### Exposure

Emissions of CNTs may, depending on the application, occur at all stages in the product life cycle: synthesis, production of intermediates, further processing, product use, and disposal. From the production sites, emission of CNTs into surrounding air depends on process control, handling procedures (yielding, bottling, packing), cleaning, safety installations and procedures (in case of leakages and accidents), and waste conditioning. These procedures may also lead to release of CNTs into the wastewater. Waste items such as contaminated hand gloves, packaging, or worn filter pads could disperse CNTs in cases of sufficient agitation. The CNTs released from the different stages may show very different characteristics such as length, surface properties, attachments, and agglomeration size. The variety of effects observed in the studies seems to result from the characteristics of the particular CNTs tested. It is therefore essential to scrutinize whether CNTs used in these studies are of the same type as the CNTs that organisms may be exposed to under real environmental conditions. A critical investigation of the types of exposures to CNTs before using CNTs in studies seems crucial for the information value of the study. Therefore, focusing on characterized exposure scenarios could be very useful when conducting toxicologic studies.

### Environmental compartments

The fate of CNTs in environmental compartments may differ depending on their specific properties such as surface chemistry, electrical properties, and oxidative potential. Studies show that functionalization and state of aggregation are likely to influence the behavior in water and soil. It would therefore be useful to characterize the CNT type and form that could be released into the environmental compartments to assess the fate of CNTs. However, the physical and chemical conditions of the different environmental compartments (such as redox potential, pH, temperature, ultraviolet light, or synergistic effects with toxins) are also likely to alter the properties of CNTs or their functional attachments, and thus their environmental fate. Other molecules or particles stick easily to the surface of CNTs and may alter the the behavior of CNTs. Consequently, pollutants may be transported in the environmental compartments. Therefore, the question arises as to whether and to what extent it would be possible to foresee and control the effects caused by different CNT material properties in the environment.

### Environmental health end points

Once CNTs are released into environmental compartments, preliminary studies have shown CNTs to be bioavailable to different organisms in addition to being biopersistent. One can therefore not exclude the possibility that CNTs may accumulate along the food chain. These studies emphasize a further need to study eco-toxicity as well as longer time-scale impacts covering bioaccumulation, biopersistency, and negative effects on reproduction.

Once taken up by humans or other species, CNTs may cause oxidative stress, inflammation, cell damage, adverse effects on cell performance, and, in a long-term perspective, pathological effects like granulomas, fibrosis, and wall thickening. These effects have been observed time and dose dependently in the majority of toxicology studies.

However, a remarkable range of results has been observed in toxicology studies (Supplemental Material, Tables S4 and S5; http://www.ehponline.org/docs/2007/9652/suppl.pdf). Comparing these different studies is difficult because different CNT species and cell model systems have been employed. An in-depth characterization of the CNTs employed is crucial for interpretation of cytotoxicologic and toxicologic assessments. The contradictions in some of the results obtained could also originate from the types and degrees of agglomeration of the CNTs. In addition, the surfactants or surface coating may have a significant influence on the CNT–cell interaction, an effect that remained unaccounted for in several studies.

Strategies to enhance the explanatory power of future toxicology studies should comprise some standardization of the CNT material, test procedures, and cell models/organisms employed. To that end, experts recommended using SWCNTs from arc discharge synthesis, as these SWCNTs show relatively good purity and structural uniformity (Supplemental Material; http://www.ehponline.org/docs/2007/9652/suppl.pdf). In addition, a set of toxic equivalency factors (TEFs) would be useful for comparing the toxicologic effects of different types of CNTs. TEFs have been developed to compare the toxicity of substance classes such as PAHs, which comprise a wide range of compounds (Nisbet and LaGoy 1992). The establishment of TEFs for CNTs would need to consider nanospecific properties of the material (e.g., particle size, aspect ratio, agglomeration state) and would require investigation of mechanisms and dose–response dependency on the cellular level.

To what extent CNTs can enter the bloodstream, for example, through the alveolar passage, and whether organisms are able to eliminate pristine CNTs remain unexplained. To date, no biodegradation mechanisms for CNTs that support elimination from the organism have been investigated. However, agglomeration and immobilization of CNTs within tissue have been observed, for example, in lung tissue. Some studies have shown that functionalization may, in addition to influencing CNT mobility, also influence the degree of toxicity. Functionalization may therefore be a key parameter for controlling the impact of CNTs on human health and the environment.

The important considerations discussed above combine different scientific disciplines ranging from materials science to biology. Integrating nanotoxicology with a life-cycle perspective will therefore be a prerequisite for the development of nanotechnology-based applications in a safe and responsible manner.
